# A Critical Evaluation of microRNA Biomarkers in Non-Neoplastic Disease

**DOI:** 10.1371/journal.pone.0089565

**Published:** 2014-02-26

**Authors:** Baqer A. Haider, Alexander S. Baras, Matthew N. McCall, Joshua A. Hertel, Toby C. Cornish, Marc K. Halushka

**Affiliations:** 1 Department of Pathology, Johns Hopkins University School of Medicine, Baltimore Maryland, United States of America; 2 Department of Biostatistics and Computational Biology, University of Rochester, Rochester, New York, United States of America; Children's National Medical Center, Washington, DC, United States of America

## Abstract

**Background:**

MicroRNAs (miRNAs) are small (∼22-nt), stable RNAs that critically modulate post-transcriptional gene regulation. MicroRNAs can be found in the blood as components of serum, plasma and peripheral blood mononuclear cells (PBMCs). Many microRNAs have been reported to be specific biomarkers in a variety of non-neoplastic diseases. To date, no one has globally evaluated these proposed clinical biomarkers for general quality or disease specificity. We hypothesized that the cellular source of circulating microRNAs should correlate with cells involved in specific non-neoplastic disease processes. Appropriate cell expression data would inform on the quality and usefulness of each microRNA as a biomarker for specific diseases. We further hypothesized a useful clinical microRNA biomarker would have specificity to a single disease.

**Methods and Findings:**

We identified 416 microRNA biomarkers, of which 192 were unique, in 104 publications covering 57 diseases. One hundred and thirty-nine microRNAs (33%) represented biologically plausible biomarkers, corresponding to non-ubiquitous microRNAs expressed in disease-appropriate cell types. However, at a global level, many of these microRNAs were reported as “specific” biomarkers for two or more unrelated diseases with 6 microRNAs (miR-21, miR-16, miR-146a, miR-155, miR-126 and miR-223) being reported as biomarkers for 9 or more distinct diseases. Other biomarkers corresponded to common patterns of cellular injury, such as the liver-specific microRNA, miR-122, which was elevated in a disparate set of diseases that injure the liver primarily or secondarily including hepatitis B, hepatitis C, sepsis, and myocardial infarction.

**Conclusions:**

Only a subset of reported blood-based microRNA biomarkers have specificity for a particular disease. The remainder of the reported non-neoplastic biomarkers are either biologically implausible, non-specific, or uninterpretable due to limitations of our current understanding of microRNA expression.

## Introduction

MicroRNAs (miRNAs) are an important class of small (∼22-nt) regulatory RNAs that are intrinsic to post-transcriptional gene control. MicroRNAs bind to the 3′UTR regions of mRNAs and either block translation or cause message degradation through RNA-induced silencing complex (RISC) mediated events [1]. Since their discovery a mere decade ago, this family of small RNA has been found to be quite common. miRBase.org, the central database and repository of microRNAs, lists 2,578 human microRNAs in its most recent version (v20.0) [2]. MicroRNAs are found throughout the genome, with transcriptional units being primarily intronic to mRNAs or in polycistronic microRNA clusters containing from 2 to 50 microRNAs. Within these genomic locations, regulatory mechanisms have arisen such that microRNAs can be ubiquitously or variably expressed in different tissues and cell types. Some microRNAs (ex. miR-126, miR-133a, miR-122, miR-451a) are known to have cell type specificity, while others remain to be characterized [3]–[6].

The normal “life-cycle” of a microRNA is to be transcribed and processed by Drosha in the nucleus, then transported to the cytoplasm to be spliced by Dicer before associating with a target mRNA in the RISC to inhibit translation [1]. In addition to these cytoplasmic and nuclear locations, microRNAs are located in other cellular and non-cellular compartments, including mitochondria, blood serum, exosomes, and microvesicles [7]–[9]. It is thought that intracellular transport of microRNAs may occur via exosomes or microvesicles.

MicroRNAs were first identified in human blood in 2008 [10]. This discovery generated enormous enthusiasm for the potential use of plasma or serum microRNAs as biomarkers of neoplastic and non-neoplastic disease. MicroRNAs are especially appealing as biomarkers because they are not prone to RNase degradation and remain stable in stored samples [11]. Thus, an enormous number of studies investigating the role of microRNAs as biomarkers of neoplastic and non-neoplastic diseases have been published in a short period of time.

The term biomarker has different meanings depending on context. Clinical biomarkers can be used as a tool for staging or classifying the extent of disease. They can predict or monitor clinical response to intervention. They can also serve to diagnose patients with a disease or abnormal condition [12]. It is this last definition for which microRNA biomarkers have primarily been used. We have reasoned that a useful microRNA clinical biomarker should, at a minimum, be expressed in a cell type involved in the specific disease process. Beyond appropriate cell localization, it has been suggested that a microRNA's expression must be altered in concert with the disease process. That is true if one is trying to use blood-based microRNAs to delineate the underlying biology, but not necessary for certain clinical uses. For example, some microRNAs and proteins (i.e. troponin T) appear in the serum/plasma as a result of cell death and the spilling of cellular contents, rather than being specifically up or down regulated in the disease process. Thus, the serum/protein presence of a uniquely expressed microRNA found in a particular cell type can inform on an injurious disease involving that cell.

Interpretation of novel microRNA blood-based biomarkers reported in recent studies is limited by our understanding of the expression patterns of specific microRNAs at the cellular level. Specifically, when a microRNA is elevated or decreased in serum or plasma, the origin of that change is unknown. It may represent altered cellular regulation of a given microRNA that is secreted into a microvesicle. It may indicate cellular injury related to the disease. The observed changes may also be unrelated to the disease in question, resulting instead from methodological problems or as a secondary phenomenon of the disease process or treatment. This last point was highlighted by a recent study by the Tewari group. They demonstrated that of 79 solid tumor circulating microRNA biomarkers reported in the literature, 47 (58%) were highly expressed in hematologic cells (red blood cells, granulocytes, platelets, monocytes, etc.) [13]. Some of these microRNA biomarkers (e.g. miR-142-3p, miR-486-5p) are not even expressed in epithelial cancer cell types. They conclude that many “biomarker” microRNAs represent increased red blood cell lysis or changing white blood cell counts, possible secondary effects of malignancy or malignancy treatment, rather than being products of the neoplasm itself.

The same factors described by Tewari *et al*. could, in theory, affect non-neoplastic microRNA biomarkers. To address this concern, we performed a critical evaluation of reported microRNA non-neoplastic disease biomarkers with the hypothesis that some reported microRNA biomarkers lack utility as they are not expressed in a cell type known to be altered/damaged in the disease, are insufficiently specific for the disease in question, or are not supported by methodologically sound evidence.

To determine useful microRNA biomarkers we performed an extensive literature and database search to identify all reports of microRNA as serum, plasma and peripheral blood mononuclear cell (PBMC) biomarkers for non-neoplastic disease. From publically-available Gene Expression Omnibus (GEO) and ArrayExpress Agilent microRNA array data, we created a novel cell-specific microRNA array expression table that allows, for the first time, the proper sourcing of microRNAs to a cell type of origin. We then evaluated the plausibility of each reported microRNA biomarker by correlating disease process with the cellular expression pattern. MicroRNA expression needed to be both cellularly relevant to the disease in question and non-ubiquitous to be considered a plausible candidate as a biomarker. Ubiquitous microRNAs, being expressed widely across cell types, will not have the specificity required to be useful biomarkers even if they are truly modified in a specific disease. We also evaluated the microRNAs in the context of the entire study set, which allowed us to not just determine their individual quality, but also their specificity to a single disease. Thus, our two-tiered approach provides both “tree” and “forest” level data. This has allowed us to comment on both “quality” and “specificity” for each biomarker.

## Methods

### Discovery of non-neoplastic microRNA serum biomarkers publications

We conducted a two-fold method of identifying non-neoplastic microRNA biomarker manuscripts. We searched PubMed using the following key terms “microRNA biomarker,” “microRNA serum biomarker,” “microRNA plasma biomarker” and “plasma miRNA.” We separately investigated every suggestive manuscript linked to from the Human MicroRNA Disease Database (HMDD) [14]. We screened 130 articles – 86 from PubMed and 44 from the HMDD (Figure S1). Of these, 26 were excluded as being reviews, animal model studies, duplicates, a stem cell study, a study of cerebral spinal fluid, or we were unable to obtain the manuscript. The discovery phase ended February 9, 2013.

### Manuscript data point collection and curation

Each manuscript was downloaded and relevant information was obtained from each. Collected data included the disease(s) studied, microRNA detection method(s), sample size, number of microRNAs examined, microRNAs reported as biomarkers, normalization strategy, submission of array data to a public repository (GEO or ArrayExpress), and journal title. Only microRNAs that were validated in two-step systems or were otherwise designated as biomarkers in the study were included in our microRNA list. This resulted in the exclusion in our study of many microRNAs found in the first pass that were not followed up in the second step. Population sizes were defined as the maximum number of all individuals used in both discovery and validation steps. Due to a range of methods, publication requirements and writing styles, the actual techniques in some manuscripts were unclear or incomplete resulting in some lost data points. Over the range of publication dates of this data set (2008–2013), the nomenclature of microRNAs has undergone several revisions. Therefore, for consistency we revised old microRNA nomenclature (ex. let-7b and let-7b*) to current nomenclature (let-7b-5p and let-7b-3p) as found in miRbase.org (release 19.0) [2].

### Obtaining publically available microRNA array datasets

Although there are multiple ways to assess the utility of a blood-based biomarker (cost, reproducibility, head-to-head comparison of an established biomarker, etc.), we chose to validate these microRNAs as biomarkers by simply determining their cellular expression patterns. A good biomarker should be expressed in a cell type implicated in the disease process and should not be ubiquitously expressed. Conversely, poor biomarkers would include microRNAs that are undetected in a disease-related cell type or that are widely expressed in multiple cell types unrelated to the disease, particularly leukocytes [13]. To evaluate the microRNA biomarkers in the 104 manuscripts, we therefore needed to understand microRNA expression at the cellular level. This finely granular approach is critical because tissue level data includes a variety of cell types (endothelial cells, inflammatory cells, etc.) that may contain many “bystander” microRNAs that are not expressed in the true cell type of interest. Inclusion of these additional microRNAs could lead to misinterpretation of expression data. Such a microRNA cell-type specific expression matrix is a resource that does not currently exist, so we created one specifically for this project.

We searched the GEO and ArrayExpress databases for all human studies that contained non-malignant, non-immortalized cell and tissue microRNA expression data, that were available as of September, 2011. This yielded 705 “normal” or “control” tissues and cell data series from 108 separate experiments. For the purposes of normalization, we focused on the version 1 (V1), V2, and V3 Agilent microRNA arrays, which were the most commonly used. These arrays contain 461, 711, and 837 valid microRNAs respectively. Of the 705 initial data series, 473 were from Agilent arrays and 356 had sufficient data for analysis and normalization. The 356 were further segmented with 111 series being from 18 cells (Table S1) and the remaining 245 (69%), were from 19 tissues. The latter group was useful for normalization (Figure S2).

### Normalizing human microRNA array data

To evaluate the normal human cellular and tissue microRNA array series, it was necessary to preprocess and combine data from several Agilent microarray platforms. We began by filtering arrays that consisted of greater than 50% missing data – these most likely resulted from non-standard protocols, experimental failure, or data corruption. The microRNA probes present on each microarray platform were mapped to standardized mirbase.org MIMAT identifiers using a key generated from multiple versions of mirbase.org that mapped the changing nomenclature back to a unifying MIMAT ID.

The full data set was normalized together using a modified version of subset quantile normalization in which the subset was defined by the microRNAs in common between all array versions [15]. Full quantile normalization assumes distributional equality across samples. Subset quantile normalization relaxes this assumption by requiring distributional equality for only a subset of probes (in the original publication this subset was chosen to be the negative control probes on the arrays). The other probes are normalized based on their relationship to the chosen subset of probes. For the microRNA microarray platforms considered here, the standard implementation of subset quantile normalization is not feasible due to the relative small number of control probes present on these arrays. Moreover, full quantile normalization is also problematic due to differences in the microRNAs targeted by each platform. Therefore, we implemented a modified version of subset quantile normalization in which the subset of probes used to perform the subset quantile normalization is the set of probes in common across all platforms. This cross-platform preprocessing approach was motivated by previous work showing that large batch effects can be introduced by combining data normalized separately [16].

To correct for systemic chip-specific effects that remained after normalization, we applied the ComBat algorithm, developed to adjust for batch effects in mRNA expression profiling [17]. ComBat uses an empirical Bayes approach to estimate and adjust for both location and scale batch effects. The adjusted values were subsequently used to create a database of cellular microRNA expression profiles (Table S2). The data set was validated by examining miR-451a and miR-126-3p levels in each cell type. Through validation, we recognized that the ductal and acinar cell data is contaminated with red blood cell and endothelial cell data due to a crude microdissection technique that captured adjacent small blood vessels [18].

### Evaluation of microRNA expression in a given cell type

Each microRNA biomarker described in any manuscript was evaluated for its expression across our range of 18 unique cell types. After normalization, expression levels ranged between 12.8 and 4.8 on a log2 expression scale (Table S2). Every value below 7.0 was clearly in the noise of the array. A comparison of literature-based reports of expression patterns of microRNAs to our data indicated we should have high confidence in values above 8.0. Thus we arbitrarily used a cutoff of 8.0 to indicate positive expression in any one sample of any given cell type.

We then classified each microRNA based on the following definitions. The first level “likely” was for microRNAs expressed in a cell type known to be involved in the disease process with some level of cellular exclusivity. We used “questionable” for any reported microRNA that was not expressed at a moderate level in any cell type for which we had information. The next level, “ubiquitous,” was used for any microRNA that was found in 7 or more different cell types which we believe makes it an unlikely marker of a specific disease process. We reserved “unlikely” for those microRNAs whose expression patterns did not match with cells known to be involved in the disease process. Finally, any reported microRNAs that we did not have data on were classified as “unknown.” We took a very inclusive approach to using the first level “likely” for microRNAs and evaluated each microRNA within the context of a disease, such that some microRNAs may be “likely” for one disease but “unlikely” in another. PBMC studies required a microRNA to be a leukocyte expressed microRNA. For studies of pregnancy-related diseases (pre-eclampsia and eclampsia), we incorporated our tissue level data, as we recognized that the miR-517 family is expressed exclusively in the placenta, but we did not have syncytiotrophoblast or cytotrophoblast cell data [19].

As an example of our assignment strategy, this is the method of categorizing 10 microRNAs in 3 studies of tuberculosis. Two studies investigated PBMCs and in these studies the category “likely” was assigned to microRNAs miR-155-5p and miR-223-3p, both known to be expressed in PBMCs. MicroRNAs miR-424-5p, miR-451a, miR-144-3p and miR-365-3p are not expressed in PBMCs, so these were categorized as “unlikely.” MicroRNAs miR-155-3p and miR-21-3p are both carrier strand (minor) microRNAs which have low expression and were categorized as “questionable.” For the third study (in serum), miR-29a-3p was categorized as “ubiquitous” as it is expressed in 16 cell types and miR-93-3p was “questionable” as it too is a carrier strand (minor) microRNA with low expression.

### Determination of normalization quality

Of the variety of normalization methods used, we decided that the evaluation of RNU6B in serum, plasma or blood NOS and the use of no normalization (including unreported methods), would be poor normalization methods (N = 28). The use of any other method including spiked-in, RNU6B in PBMCs, any human microRNAs (except miR-451a) used in normalization were included as acceptable methods (N = 76) (Table S3). This schism of the data was used to compare the frequency of likely and unlikely microRNAs in poor or acceptable normalization strategies.

### Statistics

Data was maintained in Excel 2007 (Microsoft) workbooks. T-tests and χ2 analysis was performed as needed. The database of microRNA expression across cell types was based on the raw Agilent expression data files. Data analysis was performed in the R Statistical Computing language.

## Results

### MicroRNA biomarker studies identified

Through an extensive search of PubMed and the HMDD, we identified 104 publications that contained microRNA biomarker studies performed with plasma, serum, or peripheral blood mononuclear cells (Figure S1). These studies covered 57 diseases, with most being diseases of the cardiovascular (n = 32), hepatic (n = 13) or pulmonary systems (n = 5) (Table 1). Seventeen studies were of autoimmune diseases. The studies were predominantly of serum (n = 40) and plasma (n = 40), with fewer investigating PBMCs (n = 26), or blood not otherwise specified (NOS) (n = 3). Five studies were of multiple blood compartments. Additional microRNA discovery in urine and platelets, performed in a few studies, were not included in the analysis.

**Table 1:** Studies of non-neoplastic serum, plasma or PBMC microRNAs biomarkers.

Disease	Number of studies*
**Cardiovascular**	
Myocardial infarction/injury	10
Coronary artery disease	5
Heart failure	5
Acute coronary syndrome	4
Atrial fibrillation	1
Aortic stenosis	1
Cardiac arrest	1
Hypertension	1
Hypertrophic cardiomyopathy	1
Pulmonary arterial hypertension	1
Risk of myocardial infarction	1
Viral myocarditis	1
**Liver**	
Hepatitis C	4
Hepatitis B	4
Biliary atresia	1
Cirrhosis	1
Drug-induced liver injury	1
Liver transplant rejection	1
Muscle disorder induced liver	1
**Pulmonary**	
Tuberculosis	3
Acute Pulmonary embolism	1
Chronic obstructive pulmonary disease	1
**Inflammatory bowel disease**	
Crohns Disease	2
Inflammatory bowel disease	1
Ulcerative Colitis	1
**Autoimmune**	
Multiple sclerosis	5
Systemic lupus erythematosus	4
Rheumatoid arthritis	3
Scleroderma/Systemic sclerosis	3
Graves disease	1
Pediatric systemic lupus erythematosus	1
**Other**	
Sepsis	6
Diabetes	3
Preeclampsia	3
Osteoarthritis	2
Acute kidney failure	1
Alzheimer disease	1
Amyotrophic lateral sclerosis	1
Atherosclerosis obliterans	1
Bipolar mania	1
Ectopic pregnancy	1
End stage renal disease	1
Endometriosis	1
Eosinophilic esophagitis	1
Gestational diabetes	1
Hand foot and mouth disease	1
HIV	1
Huntingtons disease	1
Intracerebral hemorrhage	1
Muscular Dystrophy	1
Naturalistic Stress	1
Parkinsons disease	1
Postmenopausal osteoporosis	1
Psoriasis Vulgaris	1
Schizophrenia	1
Traumatic brain Injury	1
Zinc Depletion	1

*Citations for these studies are in Table S6.

### Variation in microRNA detection methods

Because there is no agreed upon protocol of blood microRNA biomarker discovery, we observed a wide range of reported methods. Fifty-nine studies performed qRT-PCR exclusively, investigating an average of 4 (range 1–23) microRNAs. Forty-two studies used both a microarray/RNA-seq method of microRNA discovery with secondary qRT-PCR validation of an average of 6.7 (range 1–24) microRNAs. Three studies were exclusively microarray or RNA-seq methods (Table S4). In addition, there was a wide range of total population sizes studied (range 5–982), with a median of 69 subjects (Figure S3).

We then focused our attention on the normalization strategies of the studies, to determine what were the best and/or most common methods utilized. We found that consensus for the normalization of serum, plasma and PBMC microRNA studies does not exist. Thus, many markers were used and the quality of the normalization method used varied from study to study. Overall, spiked-in non-human microRNAs (n = 35) and RNU6B (n = 32) were the most commonly described, either alone or in conjunction with other means of normalization (Table S3). A large number of intrinsic microRNAs were also used, with miR-16 being particularly common (n = 9). For 9 studies, no normalization controls could be determined from the manuscript. This includes one study that rationalized not using controls [20] and another which rejected the controls tried [21].

### Publication quality and data sharing

As these studies encompassed such a wide range of human disease and were reported in such a diverse group of journals (n = 76), we needed a measure of journal quality. Although, by many reports it is less than ideal [22], we used Impact Factor as a surrogate for quality. The two most common journals publishing these manuscripts were PLOS One (n = 14) and Clinical Chemistry (n = 4). The average 1 year Impact Factor of the publications was 4.7 with a range of 0.936 to 14.156 (Figure S4). We also sought to determine how frequently array data was submitted in public repositories. Among the 45 array-based or RNA-seq studies, only 10 (22%) deposited their data in GEO or ArrayExpress. There was no correlation between groups that deposited their data and better journal impact factor scores (t-test Impact Factor 5.1 vs. 4.9, p>0.05).

### A novel table of human cell microRNA expression

**Figure 1:**
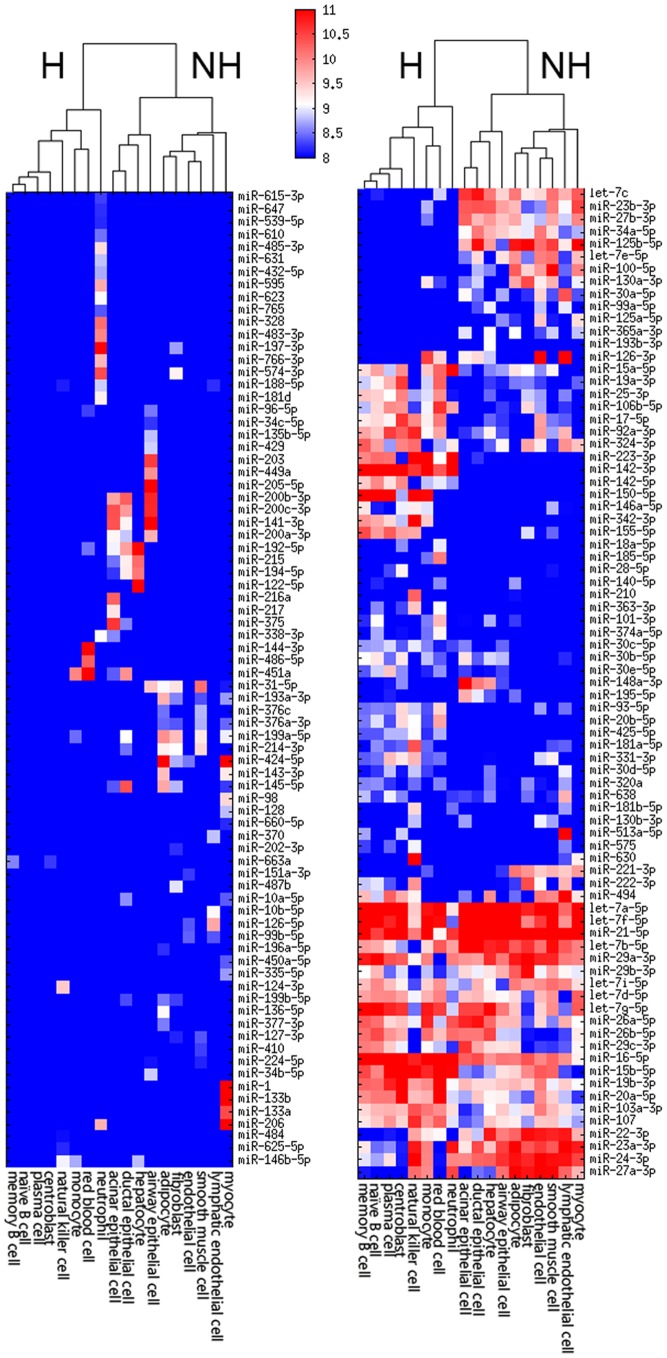
microRNA expression patterns across 18 cell types. These 157 microRNAs have variable expression patterns across the 18 cell types. Cells clustering cleanly separates hematopoetic (H) and nonhematopoetic (NH) cell types.

After extensive normalization we created a robust microRNA expression matrix organized by cell type (Figure 1 and Table S2). The collected data spans 18 cell types, reflecting a broad, but incomplete, description of most major cell types (epithelial, endothelial, mesenchymal, hematopoetic, and muscle). Our dataset nicely recapitulates known cell-specific microRNAs such as miR-1, miR-133a and miR-216 in muscle tissues and miR-122 in liver. Also, the hematopoetic cells cluster separately from the non-hematopoetic cells again consistent with known microRNA differences between these cell types [23].

### MicroRNA biomarker plausibility within a given study

Using the cellular microRNA expression matrix described above, we investigated each reported microRNA biomarker for its expression in an appropriate cell of interest. We used the five categories of potential biomarker quality: likely, questionable, ubiquitous, unlikely and unknown described in methods. We investigated 416 reported microRNA biomarkers culled from 104 separate studies. Roughly two-thirds (278) of microRNAs were reported as elevated and 142 (32%) were reported as decreased in serum, plasma or PBMCs. Of these 416 microRNAs, we scored 139 (33%) microRNAs as likely, 93 (22%) as questionable, 139 (33%) as ubiquitous, 37 (9%) as unlikely and 8 (2%) as unknown. After merging similar disease processes (ex. 10 myocardial infarction publications) together in which some microRNAs were repeatedly reported upon, 337 microRNA biomarkers remained. Of these, 96 (28%), 85 (25%), 114 (34%), 35 (10%) and 8 (2%) were likely, questionable, ubiquitous, unlikely or unknown, respectively.

Given the diverse range of categorized microRNAs, we asked whether there was any relation of these biomarker quality metrics to the method of normalization or the Impact Factor of the journal. We found no difference in the frequency of likely or unlikely calls based upon the quality of qRT-PCR normalization (χ^2^ = 0.76 and 1.17 respectively, p>0.05). There was an increase in the percentage of likely biomarkers in studies reported in journals with Impact Factors of >6 (44%) vs. studies in journals with Impact Factors up to 6 (29%) (χ^2^ =  7.47, p = 0.0063). However, for unlikely biomarkers, we observed no difference in their frequency between the quality of the higher rated (10.2%) and lower-rated (8.5%) journals (χ^2^ = 0.16, p>0.05). We determined that a closer look at this data among the more common organ systems was warranted.

### MicroRNA biomarkers in cardiac disease

**Figure 2:**
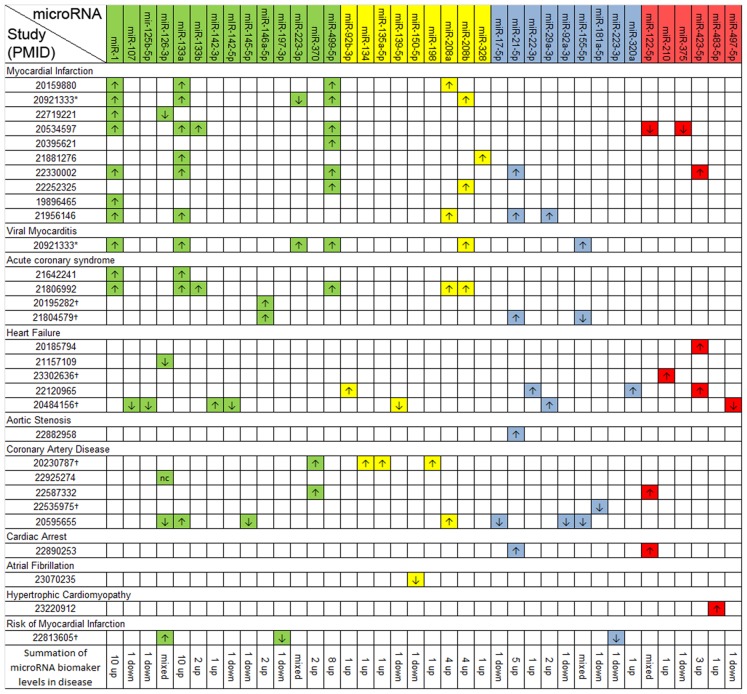
Reported microRNA biomarkers in 29 cardiac studies. Key: Study numbers  =  PMIDs; Green  =  likely; yellow  =  questionable; blue  =  ubiquitous; red  =  unlikely; * The same study investigated myocardial infarction and viral myocarditis; † These studies investigated PBMCs, not serum or plasma.

Twenty-nine studies of cardiac disease reported 87 microRNA biomarkers. Of these, 31 were unique microRNAs (Figure 2). We determined 14 (45%) to be likely, 9 (29%) to be questionable, 9 (29%) to be ubiquitous, and 6 (19%) as unlikely. The microRNAs miR-1, miR-133a, miR-133b and miR-499 are known to be highly expressed in cardiac and skeletal myocytes [6], [24]. miR-1, miR-133a and miR-133b are also expressed in breast tissue [25]. These 4 microRNAs consistently had higher blood expression across studies in which myocardial injury occurred including myocardial infarction, viral myocarditis, and acute coronary syndrome compared to normal subjects. miR-133a was also reported as a biomarker in coronary artery disease. Another 3 microRNAs, miR-21, miR-208a and miR-208b were consistently elevated across 4 studies. miR-21 is expressed in all cell types examined (Figure 1) and was therefore characterized as ubiquitous, rather than likely. miR-208a and miR-208b were characterized as questionable as we were unable to detect their signal in our cellular microRNA expression data, which included skeletal but not cardiac muscle. Four likely microRNAs, miR-107, miR-125b-5p, miR-142-3p, miR-142-5p and miR-146a-5p all failed to replicate in additional studies. miR-370, highly expressed in lymphatic endothelial cells, showed higher expression in 2 of 5 studies of coronary artery disease.

### MicroRNA biomarkers in liver disease

**Figure 3:**
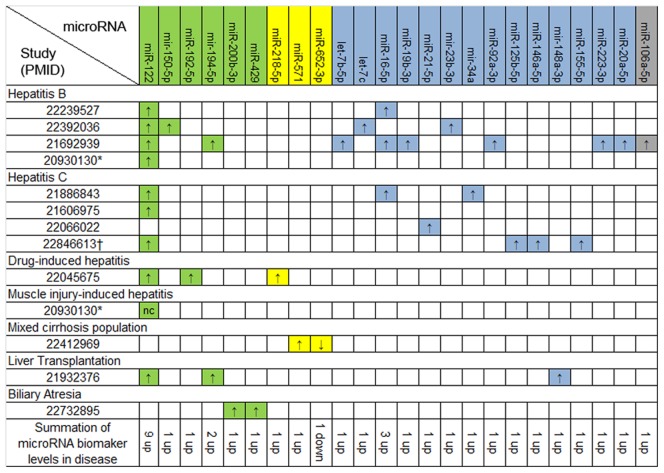
Reported microRNA biomarkers in 13 hepatic studies. Key: Study numbers  =  PMIDs; Green  =  likely; yellow  =  questionable; blue  =  ubiquitous; grey =  unknown; * The same study investigated hepatitis B and muscle injury-induced hepatitis; † Study investigated PBMCs, not just serum or plasma.

Of the 12 papers related to liver disease, 24 microRNAs were reported as biomarkers for hepatic injury (Figure 3). We determined that 6 (25%) microRNAs were likely biomarkers. Of these, only miR-122, a known liver-specific microRNA, was elevated in 9 separate studies. The majority of the reported biomarkers (n = 14), are common microRNAs with ubiquitous expression patterns. This includes miR-16 which had higher expression in 3 studies.

### MicroRNA biomarkers across different disease states

After focusing in on some of these common disease processes and finding a range of expression patterns, we investigated microRNAs across all of the studies in a “forest” level approach. In fact, many of the 416 microRNA biomarkers were described in multiple studies/diseases. After merging duplicates, we were able to collapse the list of 416 microRNA biomarkers down to 192 unique microRNAs. We plotted the 192 unique microRNAs against the 104 studies (Tables S5 and S6) to investigate overall patterns of microRNAs as biomarkers. We found that 69 (36%) microRNAs were reported in more than one manuscript. Encouragingly, there were several instances in which two or more studies of the same disease were able to replicate particular biomarkers. This was specifically true for several myocardial infarction and hepatitis microRNAs (miR-1, miR-133a, miR-499, and miR-122) as described above. However, many of the other microRNA biomarkers that were described failed to replicate in a comparable study.

One hundred and twenty-three microRNAs were described as biomarkers for a single disease. Of these, we scored 92 (75%) as questionable, ubiquitous, unlikely, or unknown biomarkers, suggesting that many of these may be spurious findings. Also adding to the complexity of finding unique biomarkers for non-neoplastic disease, we found that 47 (24%) of the 192 microRNAs reported here are also described as neoplastic biomarkers (Table S5) [13].

### MicroRNA biomarker plausibility across studies

**Figure 4:**
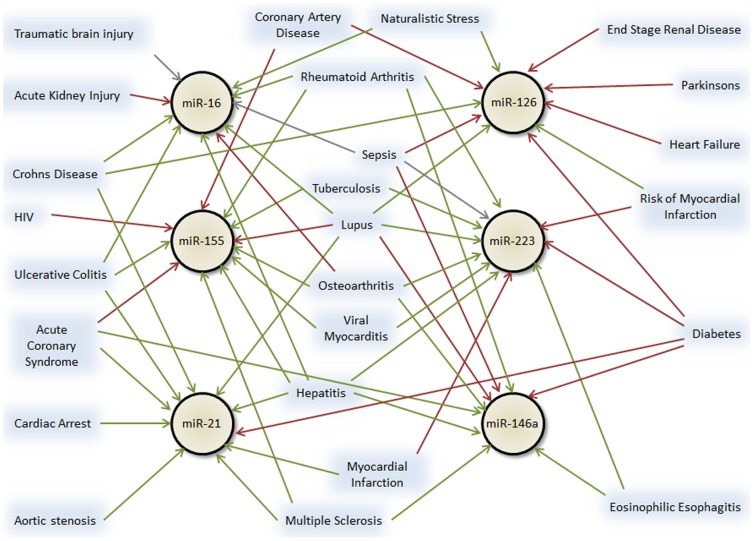
Six microRNAs have been identified as biomarkers for 9 or more diseases. Green arrows  =  higher in disease; red arrows  =  lower in disease; grey arrows  =  up or down in disease depending on the study.

As stated above, 69 microRNAs were reported in more than one publication. While several of these were in confirming studies, many microRNA biomarkers were found across two or more distinct diseases. In fact, six microRNAs (miR-16, miR-155, miR-21, miR-126, miR-223 and miR-146a) were reported as a specific biomarker in 9 or more different diseases (Figure 4). With the exception of miR-126, all of these microRNAs are highly and ubiquitously expressed across cell types (Figure 1). miR-126 is highly expressed in endothelial cells – a ubiquitous cell type in all organs - and more moderately expressed in inflammatory cells [3].

### Reproducibility of microRNAs within the same disease

Finally, we investigated how frequently a microRNA was replicated in situations where the same disease was studied more than one time. There were 15 diseases in which two or more studies were performed that could be compared. A total of 180 microRNA biomarkers were found in these 15 diseases. Of these, only 21 (12%) were replicated in two or more studies and 8 microRNAs (4%) gave opposite results between two studies.

## Discussion

This study is the first critical evaluation of microRNAs as biomarkers for non-neoplastic diseases. It differs from reviews of biomarkers that focus on a single disease entity, and fail to put the findings from one disease into the overall context of microRNAs as clinical biomarkers. This has been accomplished using data from 104 publications covering 57 different non-neoplastic diseases. We have additionally created a unique cell microRNA expression tabular matrix to evaluate the quality of each reported microRNA biomarker.

At the “tree” level we found a reasonably high percentage of likely microRNA biomarkers (33%). This was based on the microRNA possessing demonstrable specificity to a tissue type involved in the disease process. This favorable view of the microRNA as a biomarker is independent of any known biological function of the microRNA and within the cohort of 104 papers, only rarely were putative biomarkers investigated mechanistically [26]. The other common group of microRNA biomarkers was in the ubiquitous category (33%). These microRNAs are so pervasive, that a several fold change in a single affected cell type would be unlikely to affect the overall signal of the microRNA. This is similar to the idea introduced by Pritchard et al., which reasoned that a microRNA that was highly expressed in leukocytes was unlikely to be a useful biomarker of an epithelial tumor [13]. The group of questionable microRNAs (25%), those with no expression in our normal cell microRNA expression cohort, are likely of three sources. First, microRNAs could truly be upregulated in disease from a lower basal (normal) state that was not captured using normal (i.e. healthy) cell expression data. Although, these microRNAs are strong candidates as biomarkers, it is rare for a microRNA to transition from almost no expression to high expression in a non-neoplastic cell type [27]–[29].Secondly, some microRNAs are expressed in a cell type for which we lacked sufficient information. This is surely the case for miR-208a, which is known to be expressed in cardiac myocytes [30]. Finally, some of these biomarkers may have been detected at a very low level. Through questionable normalization and/or the reporting of “fold changes” without a minimum threshold of absolute expression, many of these microRNAs are detected spuriously as signal in the noise. This is undoubtedly true for most of the 23 carrier strand microRNAs (previously described as * or –as microRNAs) described as biomarkers in this cohort.

At the “forest” level, we report on several highly and ubiquitously expressed microRNAs that have been assigned as biomarkers for multiple diseases (Figure 4). This overlap can be interpreted in two ways. One is that these microRNAs truly respond non-specifically to disease stressors and are thus altered in a variety of diseases [31]. For example, miR-21 is known to be upregulated in a variety of processes including proliferative vascular disease [32], cardiac hypertrophy [33], pulmonary fibrosis [34], renal fibrosis [35] skeletal muscle injury [36] and neoplasia [37]. Also, miR-126 would be expected to be altered in any disease that causes microvascular or macrovascular damage [38]–[40]. The other option is that these microRNAs are both highly expressed and easily detected such that common methodological issues (e.g. poor normalization, variability in plasma preparation, or red blood cell lysis) could result in their repeated spurious discovery [41], [42]. Regardless, microRNAs that are altered in several disparate diseases can hardly be considered as specific clinical biomarkers for any one disease. As we learn more about higher and lower expression of microRNAs across studies, we may see clear patterns emerge. At that point, it may become useful to combine multiple microRNAs with both lower and higher expression patterns to achieve specificity for a particular disease.

Normalization and analytical methods continue to be a challenge for blood based microRNA studies. For example, RNU6B is not native to serum or plasma and is known to degrade during storage, yet it was used to normalize 19 studies in these fluids [43], [44]. Also, 9 studies (3 serum, 4 plasma and 2 PBMC) used miR-16 to normalize their data, rationalizing it was a stable microRNA. The data supporting the use of miR-16 is mixed [45], [46], with hemolysis markedly affecting miR-16 levels [47]. We point out that it was also described as a biomarker across 10 separate diseases (Figure 4), suggesting it is not a useful normalization control. Another curious method was to use miR-451a to normalize plasma data [48]. miR-451a is a red blood cell specific microRNA which, when RBCs lyse, ends up in highly variable levels in serum and plasma [13], [41]. If possible, a spiked-in non-human microRNA used at the time of RNA preparation, such as cel-miR-39, cel-miR-54 and/or cel-miR-238 [10], [47] is likely the best normalization strategy. Even that strategy can be fraught with error if the spiking is done with poor or inconsistent methodology for handling samples [42], [49]. Analytical methods on qPCR arrays were also variable with no less than 3 different global normalization methods used to evaluate the data [50].

Despite variable normalization quality, we were unable to associate better normalization procedures with an increased likelihood of obtaining likely microRNA biomarkers. We were also dismayed by the low reporting of array data depositing into a public repository (22%). This is consistent with known problems in enforcing MIAME regulations [51]. Finally, the median size of these studies (Figure S3) was only 69 subjects, which suggests that many studies were significantly underpowered to identify robust biomarkers of disease.

**Figure 5:**
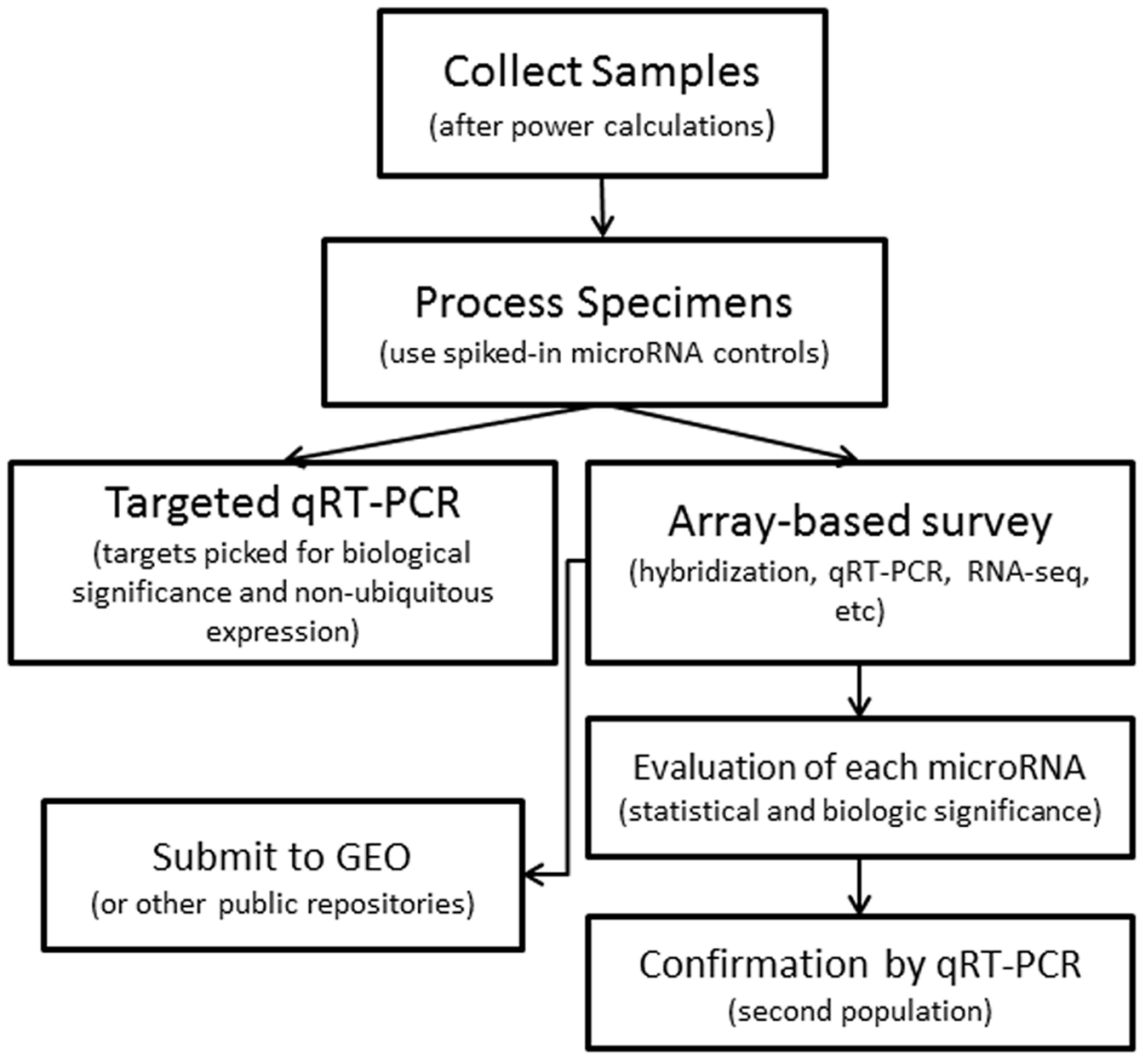
Flow diagram for proposed future microRNA biomarker studies.

We strongly believe that if there was an accessible and comprehensive database of cellular expression patterns for each microRNA, the quality of microRNAs reported as biomarkers would be vastly improved. In the 42 two-step studies (array followed by qRT-PCR), the authors attempted to replicate only a subset of all initial hits. Generally, these were the microRNAs with the highest fold expression changes. If investigators could consider both the relative change in expression and known cellular specificity, it is likely they would have chosen better microRNAs with which to follow up. Therefore, we propose a simple flow chart (Figure 5) taking ideas from the best-designed microRNA biomarker publications and incorporating cell expression localization to create an optimal method of identifying blood-based microRNA biomarkers.

We recognize some important limitations to our work. Foremost, our cellular expression data, covering only 18 cell types, was incomplete. As we showed in this study, participation in submitting to GEO or ArrayExpress is inadequate, and it is impossible to glean sufficient data from the public repositories to create a complete cell microRNA expression dataset. As we lacked certain cell types (including neurons, glial cells, cardiac myocytes and specific epithelial cell types) we undoubtedly overused our ‘questionable’ classification. Thus our lack of knowledge of cell level expression may have resulted in underreporting bonafide clinical microRNA biomarkers. Our data argues strongly for the formation of a comprehensive database of RNA-seq microRNA expression data at the cellular level. Every researcher, when faced with a number of microRNAs from an initial screen could use such a resource to make informed and rational target choices based on knowledge of their cellular expression patterns.

A second limitation of the study was that the determination of disease-associated cell type was subjective. The determination of tissues involved in each disease process was based on the consensus opinion of a board-certified pathologist and a medical school graduate. It is possible for some of the more esoteric diseases studied, that we failed to identify all cell types involved in the disease. Certainly a small percentage of microRNAs have been misassigned into some categories. Also, the inclusion of PBMC studies impacted on the categorization of some microRNAs, as PBMC studies would have to identify only microRNAs expressed in leukocytes.

Although we find that many blood based microRNA biomarkers that have been described are likely useful, a cautious evaluation of the literature is warranted. Less than a third of all reported microRNA biomarkers are expressed with some exclusivity in an appropriate diseased cell type and are not biomarkers for two or more unrelated diseases. Some microRNAs, such as miR-122 and the myocyte specific microRNAs miR-1, miR-133a, miR-499, were appropriately and consistently altered in hepatitis and myocardial infarction. However, it is best to think of these microRNAs as non-specific markers of organ injury, akin to liver function tests (i.e. AST and ALT) or troponin levels, rather than markers exclusive to a specific type of injury. Some microRNAs biomarker discoveries are encouraging, such as miR-370 which was replicated in two studies of coronary artery disease and was not reported for any other disease process.

MicroRNAs represent an exciting and explosive area of biomarker research. Over 45,000 hits on a Google Patent search for “microRNA” and “biomarker” suggest that a number of individuals anticipate this to be an important diagnostic area. Our critical evaluation of the non-neoplastic microRNA biomarkers suggests that additional rigor must be afforded to these studies to identify robust, unique and justifiable biomarkers to this wide variety of diseases.

## Supporting Information

Figure S1 **Literature search results.**
(TIF)

Figure S2 **Parsing of available microRNA data from GEO and ArrayExpress to create tissue and cell microRNA expression matrices.** A series implies array results from a single cell or tissue run.
(TIF)

Figure S3 **Histogram of study population sizes.** Population size is the maximum number of samples used across both discovery and confirmation studies (as appropriate). The median number of samples per study was 69.
(TIF)

Figure S4 **Histogram of impact factors of each journal publishing a non-neoplastic microRNA biomarker study.** The average Impact Factor across all of the journals was 4.7.
(TIF)

Table S1 **Cell specific microRNA data from which the microRNA sample array was constructed.**
(DOC)

Table S2 **Log2 normalized microRNA data for 457 microRNAs in 18 cell types.**
(XLS)

Table S3 **microRNA qRT-PCR normalization methods.**
(DOC)

Table S4 **Array, RNA-seq and qRT-PCR study designs.**
(DOC)

Table S5 **Spreadsheet of all 104 studies with their 192 unique microRNA biomarkers.** Column DG indicates the summation of cancer data. Key: PMIDs =  PubMed IDs referenced in Table S6; Green =  likely; yellow  =  questionable; blue  =  ubiquitous; red  =  unlikely; grey  =  unknown.
(XLS)

Table S6 **Cross reference of all manuscripts in the study to their PMID number.**
(XLS)

Checklist S1 (DOC)
