# Supplementary figures and images for: A Critical Evaluation of microRNA Biomarkers in Non-Neoplastic Disease
Source: PLoS One. 2014 Feb 26;9(2):e89565. doi: 10.1371/journal.pone.0089565 (PMC3935874; doi:10.1371/journal.pone.0089565)

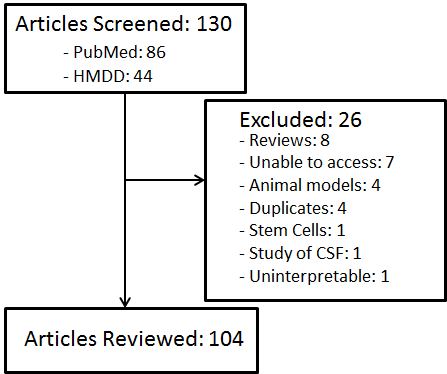

Supplement: Figure S1 — Literature search results. (TIF) [file pone.0089565.s001.tif]

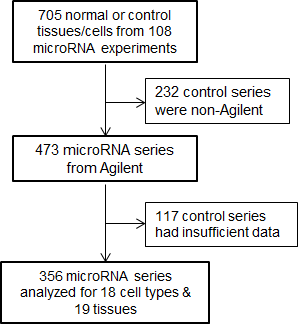

Supplement: Figure S2 — Parsing of available microRNA data from GEO and ArrayExpress to create tissue and cell microRNA expression matrices. A series implies array results from a single cell or tissue run. (TIF) [file pone.0089565.s002.tif]

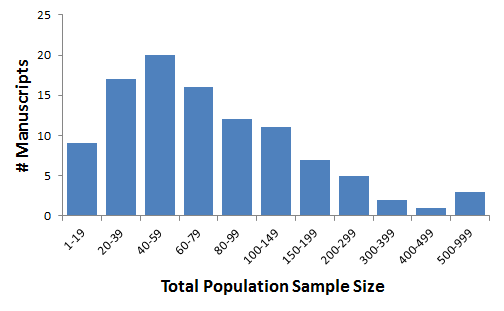

Supplement: Figure S3 — Histogram of study population sizes. Population size is the maximum number of samples used across both discovery and confirmation studies (as appropriate). The median number of samples per study was 69. (TIF) [file pone.0089565.s003.tif]

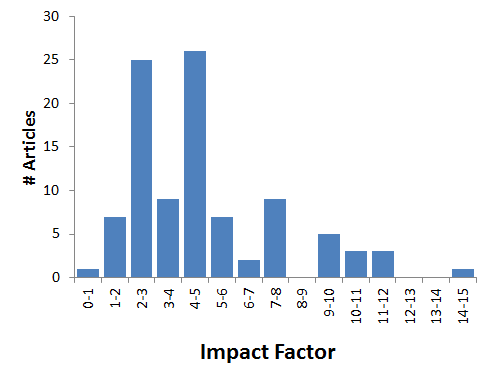

Supplement: Figure S4 — Histogram of impact factors of each journal publishing a non-neoplastic microRNA biomarker study. The average Impact Factor across all of the journals was 4.7. (TIF) [file pone.0089565.s004.tif]
