# Supplementary material for: A Critical Evaluation of microRNA Biomarkers in Non-Neoplastic Disease
Source: PLoS One. 2014 Feb 26;9(2):e89565. doi: 10.1371/journal.pone.0089565 (PMC3935874; doi:10.1371/journal.pone.0089565)
Supplement: Table S1 — Cell specific microRNA data from which the microRNA sample array was constructed. (DOC) [file pone.0089565.s005.doc]

**Supplemental Table 1**

| **Cell type** | **Number of samples** | **Agilent Array Version** |
| --- | --- | --- |
| Acinar epithelial cell | 4 | V2 |
| Adipocyte | 3 | V2 |
| Airway epithelial cell | 12 | V2 |
| Centroblast | 6 | V2 |
| Ductal epithelial cell | 4 | V2 |
| Endothelial cell | 14 | V3 |
| Fibroblast | 3 | V1 |
| Hepatocyte | 3 | V2 |
| Lymphatic endothelial cell | 8 | V1 |
| Memory B cell | 6 | V2 |
| Monocyte | 12 / 6 | V2 / V3 |
| Myocyte, skeletal | 3 | V2 |
| Naïve B cell | 6 | V2 |
| Natural Killer (NK) cell | 2 | V3 |
| Neutrophil | 11 | V2 |
| Plasma cell | 3 | V2 |
| Red blood cell | 2 | V2 |
| Smooth muscle cell | 3 | V2 |
