# Supplementary material for: A Critical Evaluation of microRNA Biomarkers in Non-Neoplastic Disease
Source: PLoS One. 2014 Feb 26;9(2):e89565. doi: 10.1371/journal.pone.0089565 (PMC3935874; doi:10.1371/journal.pone.0089565)
Supplement: Table S3 — microRNA qRT-PCR normalization methods. (DOC) [file pone.0089565.s007.doc]

**Supplemental Table 3**

|  | **Types of normalization** | **Total*** | | **Serum** | | **Plasma** | | **PBMC** | | **Blood** | |
| --- | --- | --- | --- | --- | --- | --- | --- | --- | --- | --- | --- |
| Spiked-in non-human microRNA | | 35 | 18 | | 17 | | 0 | | 0 | |  |
| RNU6B | | 32 | 10 | | 10 | | 12 | | 2 | |  |
| non-hsa-miR-16 microRNAs† | | 12 | 3 | | 7 | | 4 | | 0 | |  |
| hsa-miR-16 | | 9 | 3 | | 4 | | 2 | | 0 | |  |
| Statistical normalization‡ | | 5 | 2 | | 1 | | 2 | | 0 | |  |
| RNU48 | | 6 | 1 | | 0 | | 6 | | 0 | |  |
| Unknown / not performed | | 9 | 3 | | 5 | | 1 | | 0 | |  |
| Other § | | 9 | 3 | | 0 | | 5 | | 1 | |  |

*more than one blood fraction for some studies

PBMC = Peripheral Blood Mononuclear Cells

†let-7a-5p, miR-192-5p, miR-1249, miR-451a, miR-17-5p, miR-454-3p, miR-191-5p, miR-223-3p, miR-320b, miR-543, miR-654-3p, miR-103a-3p, miR-302a-3p, miR-372, miR-302d-3p, miR-133a

‡Median normalization, quantile normalization X2, normalization to the geometric mean and linear transformation

§5s rRNA, 18s rRNA, GAPDH, RNU5G, RNU44, RNU66, U18 snoRNA, Z30
