# Supplementary material for: A Critical Evaluation of microRNA Biomarkers in Non-Neoplastic Disease
Source: PLoS One. 2014 Feb 26;9(2):e89565. doi: 10.1371/journal.pone.0089565 (PMC3935874; doi:10.1371/journal.pone.0089565)
Supplement: Table S4 — Array, RNA-seq and qRT-PCR study designs. (DOC) [file pone.0089565.s008.doc]

**Supplemental Table 4**

| **Study design** | **Total*** | **Serum** | **Plasma** | **PBMC** | **Blood** |
| --- | --- | --- | --- | --- | --- |
| Array/RNA-seq only | 3 | 2 | 1 | 0 | 0 |
| Array and qRT-PCR | 42 | 14 | 14 | 14 | 2 |
| qRT-PCR only | 59 | 24 | 25 | 12 | 1 |

*more than one blood fraction in some studies.
